# Inhibition of Human Prostate and Bladder Smooth Muscle Contraction, Vasoconstriction of Porcine Renal and Coronary Arteries, and Growth-Related Functions of Prostate Stromal Cells by Presumed Small Molecule Gα_q/11_ Inhibitor, YM-254890

**DOI:** 10.3389/fphys.2022.884057

**Published:** 2022-05-23

**Authors:** Alexander Tamalunas, Amin Wendt, Florian Springer, Anna Ciotkowska, Beata Rutz, Ruixiao Wang, Ru Huang, Yuhan Liu, Heiko Schulz, Stephan Ledderose, Giuseppe Magistro, Christian G. Stief, Martin Hennenberg

**Affiliations:** ^1^ Department of Urology, University Hospital, LMU Munich, Munich, Germany; ^2^ Department of Pathology, University Hospital Munich, LMU Munich, Munich, Germany

**Keywords:** cardiovascular pharmacology, G proteins, lower urinary tract symptoms, prostatic hyperplasia, smooth muscle contraction, vasoconstriction, adrenergic receptor (AR)

## Abstract

**Introduction:** Lower urinary tract symptoms (LUTS) involve benign prostatic hyperplasia (BPH) and overactive bladder (OAB). Standard-of-care medical treatment includes α_1_-blockers and antimuscarinics for reduction of prostate and detrusor smooth muscle tone, respectively, and 5α-reductase inhibitors (5-ARI) to prevent prostate growth. Current medications are marked by high discontinuation rates due to unfavourable balance between efficacy and treatment-limiting side effects, ranging from dry mouth for antimuscarinics to cardiovascular dysregulation and a tendency to fall for α_1_-blockers, which results from hypotension, due to vasorelaxation. Agonist-induced smooth muscle contractions are caused by activation of receptor-coupled G-proteins. However, little is known about receptor- and organ-specific differences in coupling to G-proteins. With YM-254890, a small molecule inhibitor with presumed specificity for Gα_q/11_ became recently available. Here, we investigated effects of YM-254890 on prostate, bladder and vascular smooth muscle contraction, and on growth-related functions in prostate stromal cells.

**Methods:** Contractions of human prostate and detrusor tissues, porcine renal and coronary arteries were induced in an organ bath. Proliferation (EdU assay), growth (colony formation), apoptosis and cell death (flow cytometry), viability (CCK-8) and actin organization (phalloidin staining) were studied in cultured human prostate stromal cells (WPMY-1).

**Results:** Contractions by α_1_-adrenergic agonists, U46619, endothelin-1, and neurogenic contractions were nearly completely inhibited by YM-254890 (30 nM) in prostate tissues. Contractions by cholinergic agonists, U46619, endothelin-1, and neurogenic contractions were only partly inhibited in detrusor tissues. Contractions by α_1_-adrenergic agonists, U46619, endothelin-1, and neurogenic contractions were strongly, but not fully inhibited in renal arteries. Contractions by cholinergic agonists were completely, but by U46619 and endothelin-1 only strongly inhibited, and neurogenic contractions reduced by half in coronary arteries. YM-254890 had no effect on agonist-independent contractions induced by highmolar (80 mM) potassium chloride (KCl). Neurogenic detrusor contractions were fully sensitive to tetrodotoxin. In WPMY-1 cells, YM-254890 caused breakdown of actin polymerization and organization, and obvious, but clearly limited decreases of proliferation rate, colony formation and viability, and slightly increased apoptosis.

**Conclusion:** Intracellular post-receptor signaling pathways are shared by Gα_q_-coupled contractile receptors in multiple smooth muscle-rich organs, but to different extent. While inhibition of Gα_q/11_ causes actin breakdown, anti-proliferative effects were detectable but clearly limited. Together this may aid in developing future pharmaceutical targets for LUTS and antihypertensive medication.

## 1 Introduction

Smooth muscle contraction is essential for lower urinary tract and vascular functions and occupies a central position in pathophysiology and treatment of lower urinary tract symptoms (LUTS) and cardiovascular diseases, both belonging to the most common non-malignant diseases. LUTS consist of both voiding and storage symptoms (Oelke et al., 2009; Gravas et al., 2021). Urethral obstruction is most commonly caused as direct consequence of benign prostatic hyperplasia (BPH), leading to voiding symptoms by prostatic enlargement and increased smooth muscle tone in the hyperplastic prostate (Lepor, 2004). Storage symptoms are caused by spontaneous contractions of the detrusor muscle, referred to as overactive bladder (OAB) (Patel and Chapple, 2008; Chapple, 2011). LUTS commonly lead to considerable loss of quality of life, but also to social withdrawal and depression, while severe complications include urge incontinence, urinary retention, and infections (Chapple, 2011; Oelke et al., 2013). Storage and voiding disorders affect large parts of the population. Estimations for the year 2018 amounted the worldwide number of patients with storage symptoms to 2.7 billion, and those with voiding symptoms to 1.1 billion (Lepor, 2004). While storage symptoms predominantly include nocturia, urge incontinence, and urinary frequency, voiding symptoms most commonly consist of urinary hesitancy, intermittency, and the inability to completely empty the urinary bladder (Chapple, 2011). A considerable proportion of patients with LUTS suggestive of BPH also suffer from OAB-related symptoms, subsumed in the term “mixed LUTS” (Gravas et al., 2021). In both BPH and OAB, exaggerated smooth muscle tone and cell proliferation are important targets for medical therapy (Lepor, 2004; Oelke et al., 2013; Nambiar et al., 2018).

While activation of α_1_-adrenoceptors causes smooth muscle contraction in the prostate stroma, hyperplastic prostate stromal cell growth is facilitated through dihydrotestosterone (Lepor, 2004; Hennenberg et al., 2014). Reduction of testosterone is catalyzed by 5α-reductase (5-AR) into its biologically more active metabolite dihydrotestosterone. As α_1_-adrenoceptor antagonists (α_1_-blockers) are used for the immediate relief of LUTS by inhibiting adrenergic smooth muscle contraction, they are often administered in combination with 5α-reductase inhibitors (5-ARI) for concomitant reduction of prostate size (Oelke et al., 2013). However, α_1_-adrenoceptor antagonists improve prostate symptom scores (IPSS) and urinary flow rates (Q_max_) by no more than 50 %, and 5-ARI reduce prostate size only up to 25% after long-term use, leading to high discontinuation rates and need for definitive surgery (Michel et al., 1998; Naslund and Miner, 2007; Magistro and Stief, 2020; Tamalunas et al., 2021). Multiple studies have repeatedly suggested that adrenoceptors may promote and regulate cell proliferation and prostate growth (Kyprianou et al., 1998; Kyprianou et al., 2000; Glassman et al., 2001; Chagas-Silva et al., 2014; Nascimento-Viana et al., 2019). Based on findings from cell culture studies, animal models, and analyses of human tissues from patients being treated with α_1_-adrenoceptor antagonists, it has been suggested that α_1_-adrenoceptors may induce proliferation and suppress apoptosis (Kyprianou et al., 1998; Kyprianou et al., 2000). However, clinical studies fail to confirm the potential inhibitory effect of α_1_-blockers on prostate growth, and the European Association of Urology (EAU) guideline’s consensus clearly acknowledges, that α_1_-adrenoceptor antagonists do not reduce prostate volume (Roehrborn, 2006; Roehrborn et al., 2008; Roehrborn et al., 2010; Oelke et al., 2013).

Vascular smooth muscle contraction, in turn, is a main cause and important target for drug treatment in wide-spread cardiovascular diseases, including arterial hypertension, diabetic nephropathy, coronary artery disease and others. Worldwide numbers of annual deaths have recently been extrapolated to 7.7–10.4 million for elevated systolic blood pressure, and to 18.6 million in the context of any cardiovascular disease, including ischemic heart disease (Roth et al., 2020; Zhou et al., 2021). Diabetic nephropathy is a major complication of diabetes, affecting up to 35% of diabetic patients and accounting for 50% of patients needing dialysis or kidney transplantation in the United States (DeFronzo et al., 2021; Lin et al., 2021). Reduced renal function in diabetic nephropathy is caused by increased glomerular capillary pressure and elevated smooth muscle tone in efferent glomerular arterioles, which again are related to systemic blood pressure and hypertension (Khavandi et al., 2009; DeFronzo et al., 2021). Consequently, treatment of diabetic nephropathy includes drugs to reduce intrarenal vascular resistance and antihypertensive medications, in addition to glucose-lowering drugs (Cosentino et al., 2020). Thus, while drugs to inhibit vascular smooth muscle contraction are gold standard options in medical treatment of cardiovascular diseases (Brouwers et al., 2021), hypotensive side effects are limiting in medical treatment of voiding symptoms in BPH, where application of α_1_-blockers is a mainstay (Oelke et al., 2013).

**FIGURE 1:**
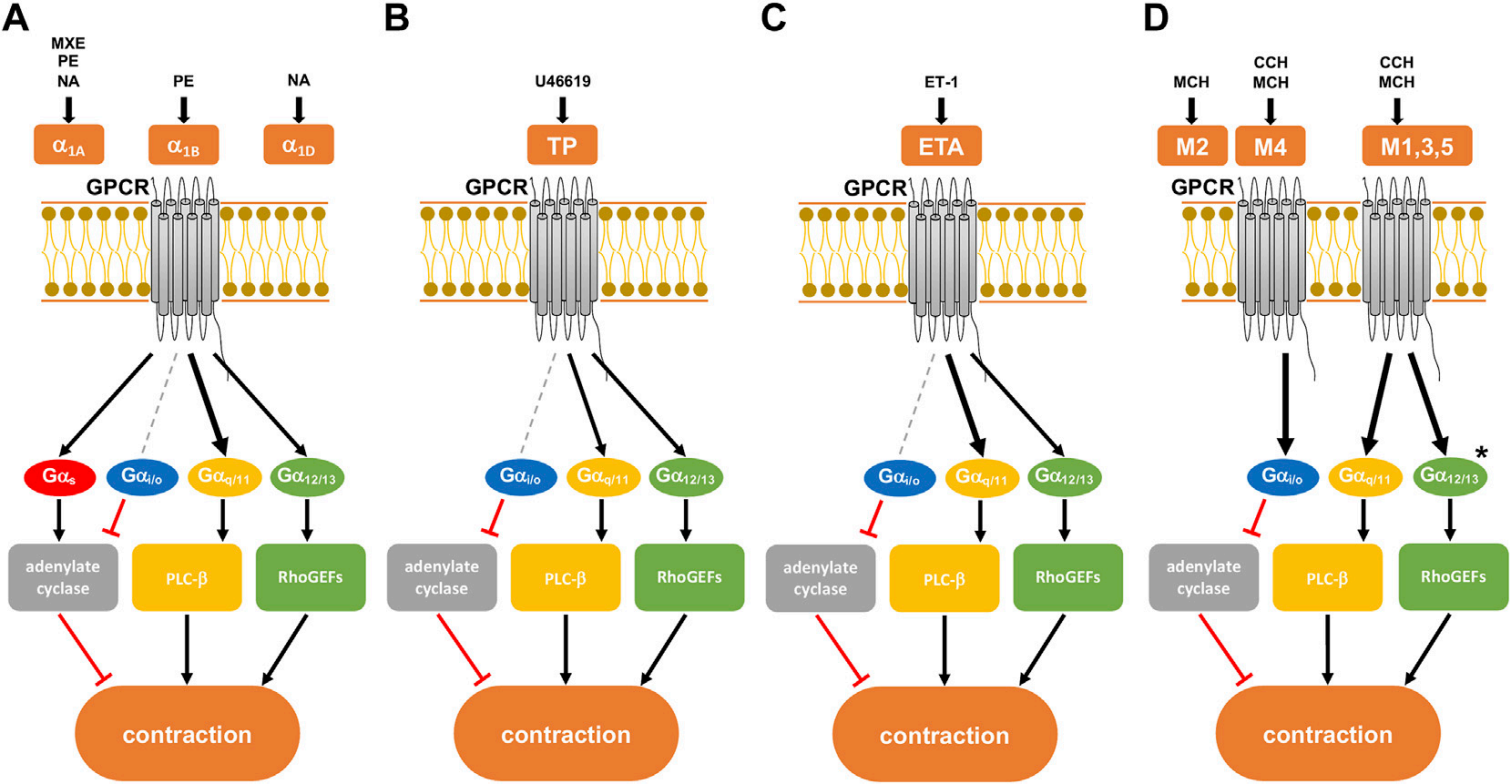
Presumed intracellular signaling pathways of G-protein coupled receptors (GPCRs) in smooth muscle contraction. GPCR activation leads to shared, but possibly also to family- or subtype-specific responses in intracellular signaling, the latter being imparted by different patterns of G protein coupling. Gαq/11 proteins may preferentially activate phospholipase (PLC)-β, leading to the production of inositol-1,4,5-trisphosphate (IP3) and diacylglycerol (DAG). Gα12/13 proteins interact with RhoGEFs and consequently preferentially active RhoA/Rho kinase. The Gαs protein family activates adenylate cyclase, while the Gαi/o protein family inhibits the enzyme. **(A)** α_1_-adrenoceptor with its subtypes (α_1A,B,D_), activated by methoxamine (MXE), phenylephrine (PE), and noradrenaline (NA). All three subtypes are present in the hyperplastic prostate at a ratio of 85:1:14 for α_1A_:α_1B_:α_1D_. **(B)** Thromboxane receptor (TP), activated by the thromboxane A2 analog U46619. **(C)** Endothelin A receptor (ETA), activated by endothelin-1 (ET-1). **(D)** Muscarinic receptor with its subtypes M1-5. All subtypes of muscarinic receptors are activated by methacholine (MCH), while carbachol (CCH) only activates subtypes M1, M3-5. Human detrusor smooth muscle predominantly contains subtypes M2 and M3 at a ratio of 3:1. After Alexander et al. (2021). Previously supposed, receptor-specific preferences in G protein coupling and intracellular receptors are indicated by different arrow sizes (bold, presumably predominant pathway for a receptor) (see text for details). * presumed signaling pathway of M3 receptors in the human detrusor.

Agonist-induced smooth muscle contractions are induced by activation of 7-transmembrane G-protein coupled receptors (GPCR) (Somlyo and Somlyo, 2000; Hennenberg et al., 2014) (Figure 1). Receptor-coupled G proteins are each composed of an *α*, *β* and *γ* subunit. Dissociation of Gα from β/γ subunits and receptors accounts for subsequent activation of intracellular post-receptor signaling, finally leading to contraction. Contractile receptors include α_1_-adrenoceptors in prostate and vascular smooth muscle, muscarinic receptors in bladder smooth muscle and coronary arteries, and thromboxane A_2_ and endothelin-1 receptors in prostate, bladder and vascular smooth muscle. All of them may be coupled to Gα_q_ or Gα_12/13_ subunits, or both (Somlyo and Somlyo, 2000; Hennenberg et al., 2014). However, only little is known about details, e.g., which receptor prefers which Gα, or whether Gα_q_ is coupled to all of them or about differences in G protein-coupling of the same receptor smooth muscle types of different organs. With YM-254890, the first small molecule inhibitor with presumed specificity for Gα_q_ has recently become available (Peng et al., 2021). To gain insight into tissue- and receptor-dependent differences in the role of Gα_q_ for agonist-induced smooth muscle contraction, and for the relevance of Gα_q_ in growth-related functions of prostate stromal cells, we examined the effect of YM-254890 on smooth muscle contractions of human prostate and detrusor tissues, on vasocontraction of porcine renal and coronary arteries, and on cellular functions of prostate stromal cells.

## 2 Materials and Methods

### 2.1 Human Tissues

Human prostate and detrusor tissues were obtained from patients who underwent radical prostatectomy for prostate cancer (*n* = 36), or radical cystectomy for bladder cancer (*n* = 39) at our tertiary referral center. While prostate tissues were obviously obtained from male patients only, detrusor tissues were obtained from male and female patients. Our research was carried out in accordance with the Declaration of Helsinki of the World Medical Association and has been approved by the ethics committee of Ludwig Maximilians University, Munich, Germany. Informed consent was obtained from all patients. All samples and data were collected and analyzed anonymously. BPH is present in approximately 80% of patients with prostate cancer (Alcaraz et al., 2009; Orsted and Bojesen, 2013). However, patients with previous transurethral resection (TURP) or holmium laser enucleation of the prostate (HoLEP) were excluded from the study. Specimens were collected immediately after surgery, followed by macroscopic examination by a pathologist. The prostate specimen was opened by a single longitudinal cut from capsule to urethra for macroscopic examination and subsequent sampling. Both intersections were checked macroscopically for any obvious tumor infiltration. Considering the fact that most prostate cancers arise in the peripheral zone, tissue samples were taken from the periurethral zone (Pradidarcheep et al., 2011; Shaikhibrahim et al., 2012). Tumor infiltration in the peri-urethral zone was very rare (<1% of prostates). For macroscopic examination and sampling of detrusor tissues, the bladder was opened by cutting from the bladder outlet to the bladder dome. Subsequently, the intravesical surface and bladder wall were checked macroscopically for tumor infiltration. Tissues were taken from the inner lateral bladder wall, provided that tumor burden in the bladder wall allowed sampling. Urothelial layers were removed from samples. Tissue samples showing tumors in the upon macroscopic inspection were not included in this study. Organ bath studies were performed immediately after sampling.

### 2.2 Porcine Arteries

Kidneys (*n* = 35) and hearts (*n* = 30) were obtained from pigs sacrificed for meat production at an age up to 12 months. Pigs were either females or castrated males. Organs were transported to a nearby butcher shop (Metzgerei Brehm, Planegg, Germany), directly following slaughter during the night (transport and temporary storage at 4°C). In the morning, organs were transferred to the laboratory. Preparation of interlobar arteries from kidneys, and of middle sections of left anterior descending arteries was started immediately. Adipose and connective tissues were removed from dissected arteries, and vessels were cut into rings, which were stored in Custodiol^®^ solution (Köhler, Bensheim, Germany) at 4°C until being used. Experiments were started within 3 hours after vessel preparation. Diameters of renal interlobar arteries ranged between 3–4 mm, and around 5 mm for coronary arteries.

### 2.3 Tension Measurements

Tissues were prepared into strips (6 mm × 3 mm × 3 mm) and mounted in 10 ml aerated (95% O_2_ and 5% CO_2_) tissue baths (Danish Myotechnology, Aahus, Denmark) with four chambers, each containing Krebs-Henseleit solution (37°C, pH 7.4) with following composition: 118 mM NaCl, 4.7 mM KCl, 2.55 mM CaCl_2_, 1.2 mM KH_2_PO_4_, 1.2 mM MgSO_4_, 25 mM NaHCO_3_, and 7.5 mM glucose. Tissue strips were stretched to 4.9 mN for human prostate and detrusor tissues, and to 10 and 20 mN for porcine renal and coronary arteries, respectively, and left to equilibrate for 45 min. In the initial phase of the equilibration period, spontaneous decreases in tone are common and warrant readjusting. Therefore, tension was adjusted three times during the equilibration period, until a stable resting tone of the respective tension was attained. After the equilibration period, maximum contraction was induced by elevation of potassium concentration to 80 mM, by addition of a 2 M potassium chloride (KCl) solution to each organ bath chamber (10 ml) in a hyperosmolar manner. Once a plateau or maximum contraction was obviously obtained, chambers were washed three times with Krebs-Henseleit solution for a total of 30 min. Subsequently YM-254890 (30 nM), tetrodotoxin (TTX, 1 µM), or equivalent amount of solvent (for controls, 100 µl DMSO for YM-254890, and 100 µl water for TTX) were added. Cumulative concentration response curves were recorded for human prostate specimens and porcine renal interlobar arteries with noradrenaline, phenylephrine, methoxamine, endothelin-1 and U46619, and for human detrusor specimens and porcine coronary arteries with methacholine, carbachol, endothelin-1 and U46619. Frequency response curves were induced by electric field stimulation (EFS), which stimulates neuronal action potentials, leading to contraction by release of endogenous neurotransmitters (Spek et al., 2021). Curves were constructed 30 min after addition test compounds or corresponding solvent. For each curve, separate control groups were performed. Effects of test compound and corresponding controls were examined in experiments using samples from the same specimen in each experiment. Within the same experiment, samples from each specimen were allocated to a control, and test compound group, so that both groups in each series had identical group sizes. Moreover, application of solvent (two chambers), and test compound (two chambers) to chambers was changed for each experiment. All values of one independent experiment were determined in duplicate, wherever this was possible. Thus, two chambers were run for controls, and two other chambers for the test compound in each experiment, if the size of sampled tissues allowed this. In experiments, where only two or three chambers could be examined due to limited amount of tissue, at least one tissue was examined with the test compound and another tissue from the same prostate with corresponding solvent. For each sample only one curve was recorded. Contractions were expressed as percentage of KCl-induced contractions for calculation of agonist-induced contractions. This accounts for the different smooth muscle content and stromal/epithelial ratios, varying degree of BPH and/or any other heterogeneity between specimen samples, and for small differences in tissue size (Kunit et al., 2014). For vessel rings, reference to KCl allows to visualize changes in receptor responsiveness, while correlations between force generation and tissue length, diameter, or weight are poor or even completely lacking in organ bath experiments (Erdogan et al., 2020). To assess the effect of YM-254890 or DMSO on KCl-induced contractions, separate series of experiments were performed for each tissue type. Contractions by KCl were induced before and after addition of YM-254890 or DMSO as described in the protocol above. After washout followed by application of YM-254890 (30 nM) or equal amount of solvent (100 µl DMSO) a second KCl-induced contraction was triggered by addition of 80 mM KCl and expressed as percentage of the first KCl-induced contraction.

### 2.4 Cell Culture

An immortalized cell line obtained from nonmalignant human prostate stroma was used for all cell culture experiments (WPMY-1) (Webber et al., 1999). Cells were obtained from American Type Culture Collection (ATCC; Manassas, VA, United States), and kept in RPMI 1640 (Gibco, Carlsbad, CA, United States) supplemented with 10% fetal calf serum (FCS) and 1% penicillin/streptomycin at 37°C with 5% CO_2_. Before addition of YM-254890, and solvent (dimethylsulfoxide, DMSO) for controls, the medium was changed to FCS-free medium. Change of medium was performed every day until cells were confluent. After cell counting and determination of proportionate volume required for further experiments, cells were transferred to culture vessels for respective experiments. For cell culture experiments, three working solutions of YM-254890 were prepared (i.e., 1, 3, and 10 µM), amounting to the final concentration of DMSO in every cell culture experiment of 10 μl/ml.

### 2.5 Cell Proliferation Assay

WPMY-1 cells were plated with a density of 30,000/well on a 16-well chambered coverslip (Thermo Scientific, Waltham, MA, United States). After 24 h, cells were treated with YM-254890, and DMSO (10 μl/ml) for controls and grown for a period of 72 h. Medium was changed to a 10 mM 5-ethynyl-2′-deoxyuridine (EdU) solution in FCS-free medium containing inhibitor or solvent following the pre-determined growth period. After another 20 h, cells were fixed with 3.7% formaldehyde. Using the “EdU-Click 555” cell proliferation assay (Baseclick, Tutzing, Germany), EdU incorporation was determined according to the manufacturer’s instructions. Incorporation of EdU into DNA is assessed by detection through fluorescing 5-carboxytetramethylrhodamine (5-TAMRA), while counterstaining of all nuclei was performed with DAPI (4′,6-diamidino-2-phenylindole). Cells were then analyzed by fluorescence microscopy (excitation: 546 nm; emission: 479 nm), and representative images of each well were taken. Thereby, each whole coverslip represents one individual experiment. Subsequently, the number of proliferating cells (i.e., EdU stained cells) was calculated in the microscopic field using ImageJ cell counter (United States National Institutes of Health, Bethesda, MD, United States). Cells are calculated individually for each experiment as a proliferation ratio (EdU stained cells divided by DAPI stained nuclei).

### 2.6 Plate Colony Assay

The ability of adherent cells to organize into colonies (>50 cells) after exposure to a specific agent can be quantified using a plate colony assay (Puck and Marcus, 1956; Rafehi et al., 2011). WPMY-1 cells were seeded in 6-well plates (100 cells/well) and incubated at 37°C for 168 h. After the initial growth period, cells were either exposed to YM-254890 (10, 30, 100 nM), or equal amount of solvent (DMSO, 10 μl/ml), and incubated for an additional period of 168 h. Cells were washed twice with phosphate-buffered saline (PBS), and fixed by 2 ml 10% trichloroacetic acid (TCA) at 4°C overnight. After that, all plates were washed five times with cold water, and stained with 0.4% sulforhodamine B (SRB) solution (diluted in 1% acetic acid) at room temperature for 30 min. Before analysis, all plates were labeled and washed by 1% acetic acid five times. Subsequently, the number of cell colonies was calculated individually for each experiment using ImageJ cell counter (United States National Institutes of Health, Bethesda, MD, United States), and compared to solvent-treated controls.

### 2.7 Cell Viability Assay

The effect of YM-254890 on cell viability was assessed using the Cell Counting Kit-8 (CCK-8) (Sigma-Aldrich, St. Louis, MO, United States). WPMY-1 cells were grown in 96-well plates (50,000 cells/well) for 24 h, before YM-254890 (10, 30, and 100 nM) or equal amount of solvent (DMSO, 10 μl/ml) for controls were added. For each concentration and time, series of *n* = 5 independent experiments were performed. Subsequently, cells were grown for a period of 24, 48, and 72 h. After the respective growth period, 10 µl of 2-(2-methoxy-4-nitrophenyl)-3-(4-nitrophenyl)-5-(2,4-disulfophenyl)-2H-tetrazoliummono sodium salt (WST-8) from CCK-8 were added, and absorbance (optical density, OD) in each well was measured at 450 nm after incubation for 120 min at 37°C. Viability of cells was reflected by optical density.

### 2.8 Flow Cytometry Analysis for Apoptosis and Cell Death

A flow cytometry-based annexin V allophycocyanin (APC) and 7-aminoactinomycin D (7-AAD) apoptosis detection kit (BD Biosciences, Franklin Lakes, NJ, United States) was used to detect cells in apoptosis (annexin V-positive, 7-AAD-negative) and dead cells (annexin V-positive, 7-AAD-positive). WPMY-1 cells were seeded in 6-well plates (250,000 cells/well) and cultured for 24 h. After addition of YM-254890, and DMSO (10 μl/ml) for controls, cells were incubated for 72 h. Subsequently, cells were washed with phosphate-buffered saline (PBS) and resuspended in annexin V binding buffer (BD Biosciences), followed by addition of 5 μl APC annexin V and 5 μl 7-AAD reagent to each sample. After incubation in the dark for 15 min at room temperature, 400 μl binding buffer were added to each sample before analysis by flow cytometry.

### 2.9 Phalloidin Staining

For fluorescence staining with phalloidin, WPMY-1 cells were plated with a density of 30,000/well on a 16-well chambered coverslip (Lab-Tek chamber slides, Thermo Scientific, Waltham, MA, United States). Cells were grown for 24 h. Cells were then treated with either YM-254890 (10, 30, and 100 nM), or equal amounts of solvent (DMSO, 10 μl/ml) for controls. Cells were then grown a subsequent period of 72 h. Staining was performed using 100 μM fluorescein isothiocyanate (FITC)-labeled phalloidin (Sigma-Aldrich, Munich, Germany), according to the manufacturer’s instruction, while counterstaining of all nuclei was performed with DAPI (4′,6-diamidino-2-phenylindole). Labeled cells were analyzed using a laser scanning microscope (Leica SP8 AOBS WLL, Wetzlar, Germany).

### 2.10 Data and Statistical Analysis

Data in frequency and concentration response curves are means ± standard deviation (SD), which are presented together with the indicated number (*n*) of independent experiments. E_max_ values and data from cell culture are presented as single values (means of two technical replicates from double determination, wherever this was possible) from each independent experiment, together with means in scatter plots. Although effect sizes become obvious from frequency and concentration response curves, some effects are additionally reported in the text. Effects of frequency/concentration response curves are reported as relative percent (%) inhibition (ratio) for all frequencies and agonist concentrations, together with 95% confidence intervals (CIs). These were calculated by referring tensions in the presence of YM-254890 to the mean of contractions in controls of the same series, at each single frequency and agonist concentration and in each single experiment, which were then summarized as mean difference (MD) with 95% CI of all frequencies, agonist concentrations and experiments in each series. E_max_ values are reported without normalization to controls as absolute mean difference (MD) with 95% CI. Data from cell culture are reported as means with 95% CIs and without normalization (EdU, colony formation, phalloidin), partly together with the mean difference (MD) and 95% CI (phalloidin), or as % inhibition together with 95% confidence intervals (CIs) (CCK-8), which were calculated by setting OD values from controls to 100% in each single experiment, or as fold of controls, which were calculated by setting values from controls to 100% in each single experiment (flowcytometry). All statistical analyses were performed using GraphPad Prism Version 9.3.0 (GraphPad Software Inc., San Diego, CA, United States). Comparison of whole frequency/concentration response curves was performed by two-way analysis of variance (ANOVA). Comparisons of contractions at single frequencies or agonist concentrations within curves by multiple comparison (after two-way ANOVA) were not included, owing to the two-dimensional character and as this is discouraged by the “GraphPad Statistics Guide”. E_max_ values were compared by a paired Student’s *t*-test. Multiple comparisons in data sets including a control group and more than one concentration of YM-254890 in cell culture experiments were performed by one-way ANOVA with Dunnett’s tests. Curve fitting was performed using GraphPad Prism 9.3.0 and limited to calculation of E_max_ values in organ bath experiments, as calculation of EC_50_ values was mostly inappropriate (in particular for YM-254890 groups), and limited to calculation of IC_50_ values in those series of cell culture experiments, where maximum effects were obviously attained in the applied concentration range. The present study and analyses show an exploratory design and were not designed to test a pre-specified statistical null hypothesis (Michel et al., 2020). Besides a lacking hypothesis, typical features of a strictly hypothesis-testing study design were lacking in our study, including a clear preset study plan, blinding, or biometric calculation of groups sizes. Consequently, *p* values reported here need to be considered as descriptive, but not as hypothesis-testing. In line with recent recommendations, the focus was on effect sizes and *p* values were used sparingly (Michel et al., 2020). Minimum numbers of experiments and group sizes for each series were pre-planned as *n* = 5/group, to allow the calculation of descriptive *p* values. Data were analyzed, after five or more experiments were performed for a given series. Subsequently, the series was discontinued if it became obvious that no effect could be expected on this basis, or if *p* values were <0.05 after comparison of frequency/concentration response curves. If these initial results were inconclusive, i.e., pointed to a possible drug effect but without *p* values <0.05, series were continued and analyzed again. This procedure was possible due to the explorative character, and as long as it is reported in detail (Michel et al., 2020). Flexible group sizes have been in fact recommended for experimental design and analysis in experimental pharmacology, if data are characterized by large variations, which applies here (Curtis et al., 2015; Curtis et al., 2018). However, interim analyses were limited to frequency and concentration response curves and did not include E_max_ values, which were calculated only after completion of series. Thus, all groups being subjected to statistical analyses were based on five or more independent experiments or included tissues from five or more patients, and the minimum group size of all groups subjected to statistical tests was *n* = 5. Moreover, all groups being compared with each other by statistical tests showed identical group sizes; consequently, any statistical comparisons between groups of different sample sizes, or between groups composed with tissues from different samples were not performed. No data or experiments were excluded from analyses.

### 2.11 Materials, Drugs and Nomenclature

YM-254890 ((R)-1-((3S,6S,9S,12S,18R,21S,22R)-21-acetamido-18-benzyl-3-((R)-1-methoxy ethyl)-4,9,10,12,16,22-hexamethyl-15-methylene-2,5,8,11,14,17,20-heptaoxo-1,19-dioxa-4,7, 10,13,16-pentaazacyclodocosan-6-yl)-2-methylpropyl (2S,3R)-2-acetamido-3-hydroxy-4-me-thylpentanoate) is an inhibitor of Gα_q/11_, and was purchased from Tocris Biosciences (Minneapolis, MN, United States) (Takasaki et al., 2004; Peng et al., 2021). YM-254890 was stored at −20°C and stock solutions were freshly prepared with DMSO before each experiment, with concentrations allowing administration of DMSO in an amount of 10 μl/ml in all samples, including controls. Noradrenaline (4-[(1R)-2-Amino-1-hydroxyethyl]-1,2-benzenediol), phenyl-ephrine ((R)-3-[-1-hydroxy-2-(methylamino)ethyl] phenol), methoxamine (α-(1-Aminoethyl)-2,5-dimeth-oxy-benzyl alcohol) are agonists for α_1_-adrenoceptors. Carbachol (carbamoylcholin; (2-Hydroxyethyl)-trimethylammonium-chlorid-carbamat) and metha-choline (2-Acetoxypropyl) trimethylam-moniumchlorid, Acetyl-β-methylcholin-chlorid) are muscarinic acetylcholine receptor agonists (Pei et al., 1998; Alexander et al., 2021). U46619 ((Z)-7-[(1-S,4R,5R,6S)-5-[(E,3S)-3-hydroxyoct-1-enyl]-3-oxabicyclo[2.2.1] heptan-6-yl]hept-5-enoic acid) is an analogue of thromboxane A_2_ and frequently used as an agonist for thromboxane receptors. Endothelin-1 is a 21-amino acid peptide with high affinity to the endothelin A (ET_A_) and B (ET_B_) receptors. Aqueous stock solutions of noradrenaline, phenylephrine, methoxamine, methacholine, and carbachol were freshly prepared before each experiment. Stock solutions of U46619 were prepared in ethanol and stock solutions of endothelin-1 in water, and both stored at −80°C until use. YM-254890 was obtained from Tocris (Bristol, United Kingdom), noradrenaline, phenylephrine, methoxamine, methacholine, and carbachol were obtained from Sigma (Munich, Germany), and U46619 and endothelin-1 from Enzo Life Sciences (Lörrach, Germany).

## 3 Results

### 3.1 Effects of YM-254890 on Contraction of Human Prostate Tissues

#### 3.1.1 Adrenergic Contractions

**FIGURE 2:**
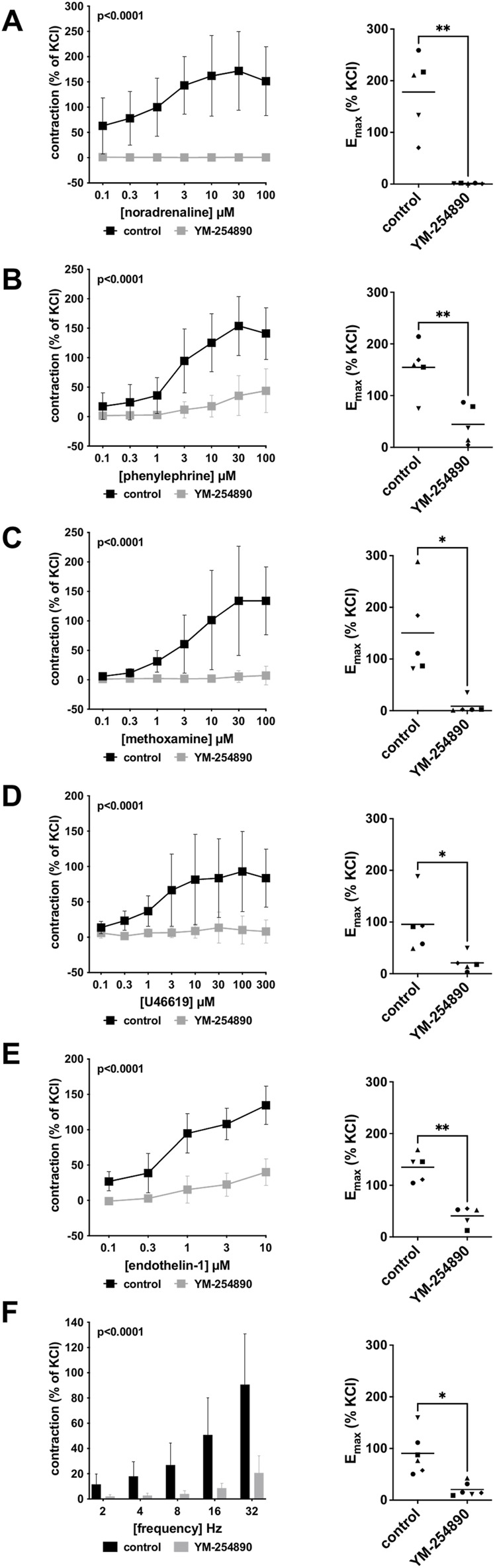
Effects YM-254890 on adrenergic and non-adrenergic, and EFS-induced neurogenic contractions of human prostate tissues. Adrenergic contractions were induced by the α1-adrenoceptor agonists noradrenaline **(A)**, phenylephrine **(B)**, and methoxamine **(C)**, while non-adrenergic contractions were induced by thromboxane A2 analog U46619 **(D)** and (Continued) endothelin-1 **(E)**, and neurogenic contraction was induced by EFS **(F)**, after addition of YM-254890 (30 nM) or equal amount of solvent (DMSO) for controls (10 μl/ml). To eliminate heterogeneities due to individual variations, different degree of BPH, varying smooth muscle content or other confounders, tensions have been expressed as percentages (%) of contraction by highmolar KCl (80 mM), being assessed before application of YM-254890 or DMSO. Each experiment used strips from different patients and data are presented as means ± SD from series with tissues from *n* = 5 patients for noradrenaline **(A)**, *n* = 5 patients for phenylephrine **(B)**, *n* = 5 patients for methoxamine **(C)**, *n* = 5 patients for U46619 **(D)**, *n* = 5 patients for endothelin-1 **(E)**, and *n* = 6 patients for EFS **(F)**. Tissue from each patient was allocated to the control and drug group examined in the same experiment, resulting in paired groups and identical group sizes in each diagram. Overall *p* values reflect comparison in two-way ANOVA between treatment and control groups (*p* values for whole groups in inserts). All single Emax values from experiments are shown in scatter plots next to their corresponding agonist-response curves (*p < 0.05).

Human prostate smooth muscle contraction was induced by the adrenergic agonists noradrenaline, methoxamine and phenylephrine following incubation with YM-254890 (30 nM), or DMSO for controls. YM-254890 inhibited noradrenaline-induced contractions by 99% (99–100) at concentrations of 0.1–100 µM (overall *p* < 0.0001 for YM-254890 vs. control; Figure 2A). E_max_ was reduced by YM-254890, amounting to 178% (84–272) of KCl-induced contractions in controls and to 1.4% (0.6–2.1) of KCl-induced contractions after application of YM-254890 [absolute MD 177% (84–270), *p* = 0.006]. Phenylephrine-induced contractions were inhibited by 85% (77–92) at concentrations of 0.1–100 µM (overall *p* < 0.0001 for YM-254890 vs. control; Figure 2B). E_max_ was reduced by YM-254890, amounting to 155% (92–217) of KCl-induced contractions in controls and to 44% (−2–91) of KCl-induced contractions after application of YM-254890 [absolute MD 110% (45–175), *p* = 0.009]. YM-254890 inhibited methoxamine-induced contractions by 91% (85 to 98) at concentrations of 0.1–100 µM (overall *p* < 0.0001 for YM-254890 vs. control; Figure 2C). E_max_ was reduced by YM-254890, amounting to 150% (42–259) of KCl-induced contractions in controls and to 9% (10–27) of KCl-induced contractions after application of YM-254890 [absolute MD 142% (23–260), *p* = 0.029].

#### 3.1.2 Non-Adrenergic Contractions

YM-254890 significantly inhibited contractions induced by U46619 by 83% (73–94) at concentrations of 0.1–300 µM (overall *p* < 0.0001 for YM-254890 vs. control; Figure 2D). E_max_ was reduced by YM-254890, amounting to 96% (27–165) of KCl-induced contractions in controls and to 21% (−1 to 43) of KCl-induced contractions after application of YM-254890 [absolute MD 75% (26–123), *p* = 0.013]. Endothelin-1-induced contractions were inhibited by 86% (75–97) at concentrations of 0.1–10 µM (overall *p* < 0.0001 for YM-254890 vs. control; Figure 2E). E_max_ was reduced by YM-254890, amounting to 135% (102–168) of KCl-induced contractions in controls and to 41% (18–64) of KCl-induced contractions after application of YM-254890 [absolute MD 94% (48–141), *p* = 0.005].

#### 3.1.3 Electric Field Stimulation-Induced Contractions

Using EFS, we investigated the effect of YM-254890 on neurogenic contractions. YM-254890 reduced EFS-induced contractions by 83% (80–85) at frequencies of 2–32 Hz (overall *p* < 0.0001 for YM-254890 vs. control; Figure 2F). E_max_ was reduced by YM-254890, amounting to 91% (48–133) of KCl-induced contractions in controls and to 21% (7–35) of KCl-induced contractions after application of YM-254890 [absolute MD 70% (20–120), *p* = 0.016].

### 3.2 Effects of YM-254890 on Contraction of Human Detrusor Tissues

#### 3.2.1 Cholinergic Contractions

**FIGURE 3:**
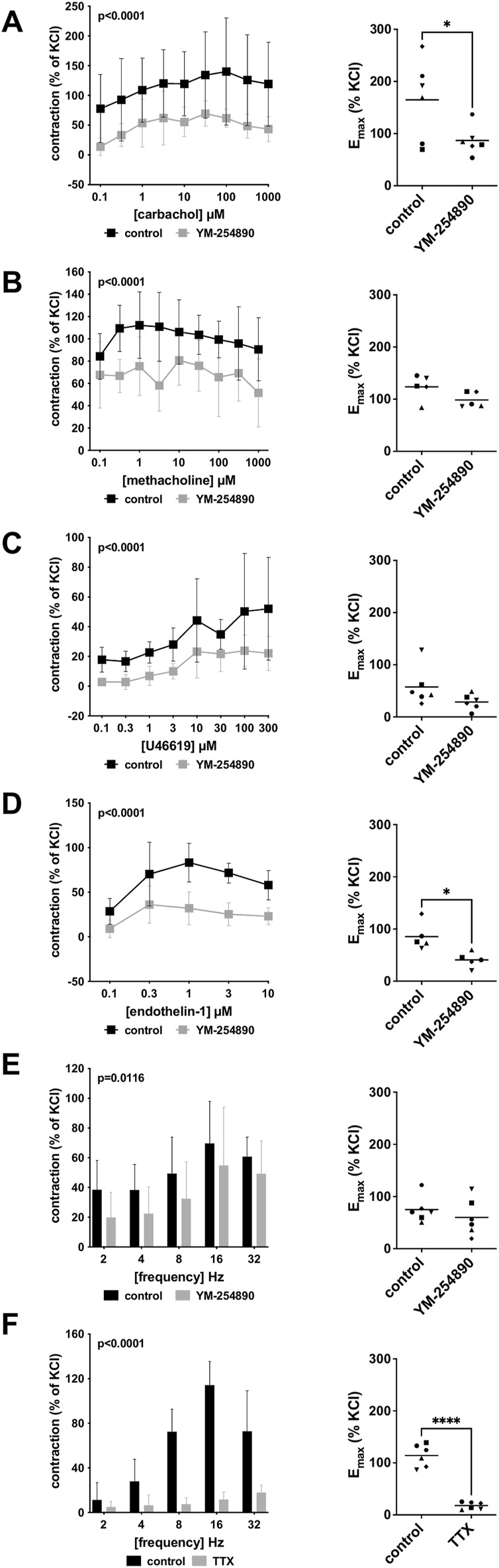
Effects of YM-254890 on cholinergic and non-cholinergic, and EFS-induced neurogenic contractions of female and male human detrusor tissues. Cholinergic contractions were induced by the m-cholinreceptor agonists carbachol **(A)**, and methacholine **(B)**, while non-cholinergic contractions were induced by thromboxane A2 analog (Continued) U46619 **(C)** and endothelin-1 **(D)**, and neurogenic contraction was induced by EFS **(E,F)**, after addition of YM-254890 (30 nM) or equal amount of solvent (DMSO) for controls (10 μl/ml), and tetrodotoxin (TTX, 1 µM) or purified water for controls. To eliminate heterogeneities due to individual variations, or varying smooth muscle content or other heterogeneities, tensions have been expressed as percentages (%) of contraction by highmolar KCl (80 mM), being assessed before application of YM-254890 or DMSO. Each experiment used strips from different patients and data are presented as means ± SD from series with tissues from *n* = 2 female and *n* = 4 male patients for carbachol **(A)**, *n* = 2 female and *n* = 3 male patients for methacholine **(B)**, *n* = 6 male patients for U46619 **(C)**, *n* = 5 male patients for endothelin-1 **(D)**, and *n* = 2 female and *n* = 4 male patients for EFS **(E)** after incubation with YM-254890, and *n* = 4 female and *n* = 2 male patients for EFS **(F)** after incubation with TTX. Tissue from each patient was allocated to the control and drug group examined in the same experiment, resulting in paired groups and identical group sizes in each diagram. Overall *p* values reflect comparison in two-way ANOVA between treatment and control groups (*p* values for whole groups in inserts). All single Emax values from experiments are shown in scatter plots next to their corresponding agonist-response curves (*p < 0.05, ****p < 0.0001).

Human detrusor smooth muscle contraction was induced by the cholinergic agonists carbachol and methacholine following incubation with YM-254890 (30 nM), or DMSO for controls. Carbachol-induced contraction was inhibited by 58% (49–66) at concentrations of 0.1–1,000 µM (overall *p* < 0.0001 for YM-254890 vs. control; Figure 3A). E_max_ was reduced by YM-254890, amounting to 165% (84–245) of KCl-induced contractions in controls and to 87% (58–116) of KCl-induced contractions after application of YM-254890 [absolute MD 78% (6–150), *p* = 0.039]. YM-254890 only partly reduced methacholine induced contraction by 32% (24–40) at concentrations of 0.1–1,000 µM (overall *p* < 0.0001 for YM-254890 vs. control Figure 3B). E_max_ remained unchanged by YM-254890, amounting to 124% (94–154) of KCl-induced contractions in controls and to 99% (81–117) of KCl-induced contractions after application of YM-254890 [absolute MD 25% (−9 to 59), *p* = 0.111].

#### 3.2.2 Non-Cholinergic Contractions

YM-254890 inhibited non-cholinergic contractions induced by U46619 by 63% (49–76) at concentrations of 0.1–300 µM (overall *p* < 0.0001 for YM-254890 vs. control; Figure 3C). E_max_ remained unchanged by YM-254890, amounting to 58% (19–96) of KCl-induced contractions in controls and to 29% (13–44) of KCl-induced contractions after application of YM-254890 [absolute MD 29% (−9 to 67), *p* = 0.111]. Endothelin-1-induced contractions were inhibited by 61% (54–67) at concentrations of 0.1–10 µM (overall *p* < 0.0001 for YM-254890 vs. control; Figure 3D). E_max_ was reduced by YM-254890, amounting to 85% (53–118) of KCl-induced contractions in controls and to 41% (23–59) of KCl-induced contractions after application of YM-254890 [absolute MD 45% (8–81), *p* = 0.027].

#### 3.2.3 Electric Field Stimulation-Induced Contractions

Using EFS, we investigated the effect of YM-254890 on neurogenic contractions of human detrusor tissue. YM-254890 reduced EFS-induced contractions by 33% (22–44) at frequencies of 2–32 Hz (overall *p* = 0.0116 for YM-254890 vs. control; Figure 3E). E_max_ remained unchanged by YM-254890, amounting to 75% (49–101) of KCl-induced contractions in controls and to 60% (23–97) of KCl-induced contractions after application of YM-254890 [absolute MD 15% (−32 to 62), *p* = 0.447]. TTX reduced EFS-induced contractions by 77% (66–89) at frequencies of 2–32 Hz (overall *p* < 0.0001 for TTX vs. control; Figure 3F). E_max_ was reduced by TTX, amounting to 114% (92–137) of KCl-induced contractions in controls and to 18% (11–25) of KCl-induced contractions after application of TTX [absolute MD 96% (75–118), *p* < 0.0001].

### 3.3 Effects of YM-254890 on Contraction of Porcine Coronary Arteries

#### 3.3.1 Cholinergic Contractions

**FIGURE 4:**
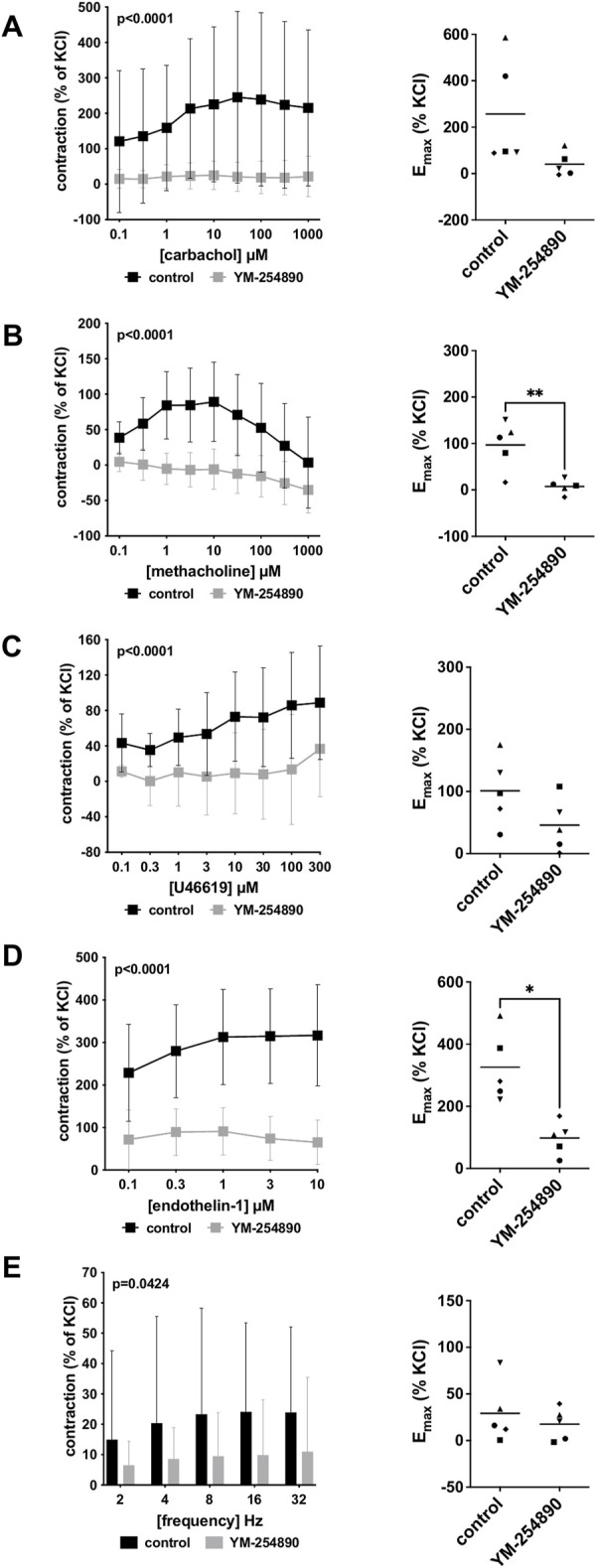
Effects of YM-254890 on cholinergic and non-cholinergic, and EFS-induced neurogenic contractions of porcine coronary artery tissues. Cholinergic contractions were induced by the m-cholinreceptor agonists carbachol **(A)**, and methacholine **(B)**, while non-cholinergic contractions were induced by thromboxane A2 analog U46619 **(C)** and endothelin-1 **(D)**, (Continued) and neurogenic contraction was induced by EFS **(E)**, after addition of YM-254890 (30 nM) or equal amount of solvent (DMSO) for controls (10 μl/ml). To eliminate heterogeneities due to individual variations, or varying smooth muscle content or other heterogeneities, tensions have been expressed as percentages (%) of contraction by highmolar KCl (80 mM), being assessed before application of YM-254890 or DMSO. Each experiment used strips from different animals and data are presented as means ± SD from series with tissues from *n* = 5 animals for carbachol **(A)**, *n* = 5 animals for methacholine **(B)**, *n* = 5 animals for U46619 **(C)**, *n* = 5 animals for endothelin-1 **(D)**, and *n* = 5 animals for EFS **(E)** after incubation with YM-254890 or DMSO. Tissue from each animal was allocated to the control and drug group examined in the same experiment, resulting in paired groups and identical group sizes in each diagram. Overall *p* values reflect comparison in two-way ANOVA between treatment and control groups (*p* values for whole groups in inserts). All single Emax values from experiments are shown in scatter plots next to their corresponding agonist-response curves (*p < 0.05).

Vascular smooth muscle contraction in porcine coronary arteries was induced by the cholinergic agonists carbachol and methacholine following incubation with YM-254890 (30 nM), or DMSO for controls. Carbachol-induced contraction was inhibited by 90% (88–91) at concentrations of 0.1–1,000 µM (overall *p* < 0.0001 for YM-254890 vs. control; Figure 4A). E_max_ remained unchanged by YM-254890, amounting to 257% (−32 to 545) of KCl-induced contractions in controls and to 41% (−24 to 105) of KCl-induced contractions after application of YM-254890 [absolute MD 216% (−42 to 474), *p* = 0.081]. YM-254890 reduced methacholine induced contraction by 118% (91–144) at concentrations of 0.1–1,000 µM (overall *p* < 0.0001 for YM-254890 vs. control; Figure 4B) resulting in tissue relaxation. E_max_ was reduced by YM-254890, amounting to 97% (33–161) of KCl-induced contractions in controls and to 7% (−12 to 26) of KCl-induced contractions after application of YM-254890 [absolute MD 90% (42–138), *p* = 0.007].

#### 3.3.2 Non-Cholinergic Contractions

YM-254890 inhibited contractions induced by U46619 by 86% (75–97) at concentrations of 0.1–300 µM (overall *p* < 0.0001 for YM-254890 vs. control; Figure 4C). E_max_ remained unchanged by YM-254890, amounting to 101% (33–170) of KCl-induced contractions in controls and to 46% (−7 to 99) of KCl-induced contractions after application of YM-254890 [absolute MD 55% (−15 to 126), *p* = 0.095]. Endothelin-1-induced contractions were inhibited by 73% (68–77) at concentrations of 0.1–10 µM (overall *p* < 0.0001 for YM-254890 vs. control; Figure 4D). E_max_ was reduced by YM-254890, amounting to 326% (187–465) of KCl-induced contractions in controls and to 98% (32–164) of KCl-induced contractions after application of YM-254890 [absolute MD 228% (76 to 381), *p* = 0.014].

#### 3.3.3 Electric Field Stimulation-Induced Contractions

Using EFS, we investigated the effect of YM-254890 on neurogenic contractions of porcine coronary arteries. YM-254890 reduced EFS-induced contractions by 57% (56–59) at frequencies of 2–32 Hz (overall *p* = 0.0424 for YM-254890 vs. control; Figure 4E). E_max_ remained unchanged by YM-254890, amounting to 29% (−11 to 70) of KCl-induced contractions in controls and to 18% (−4 to 39) of KCl-induced contractions after application of YM-254890 [absolute MD 12% (−29 to 52), *p* = 0.471].

### 3.4 Effects of YM-254890 on Contraction of Porcine Renal Arteries

#### 3.4.1 Adrenergic Contractions

**FIGURE 5:**
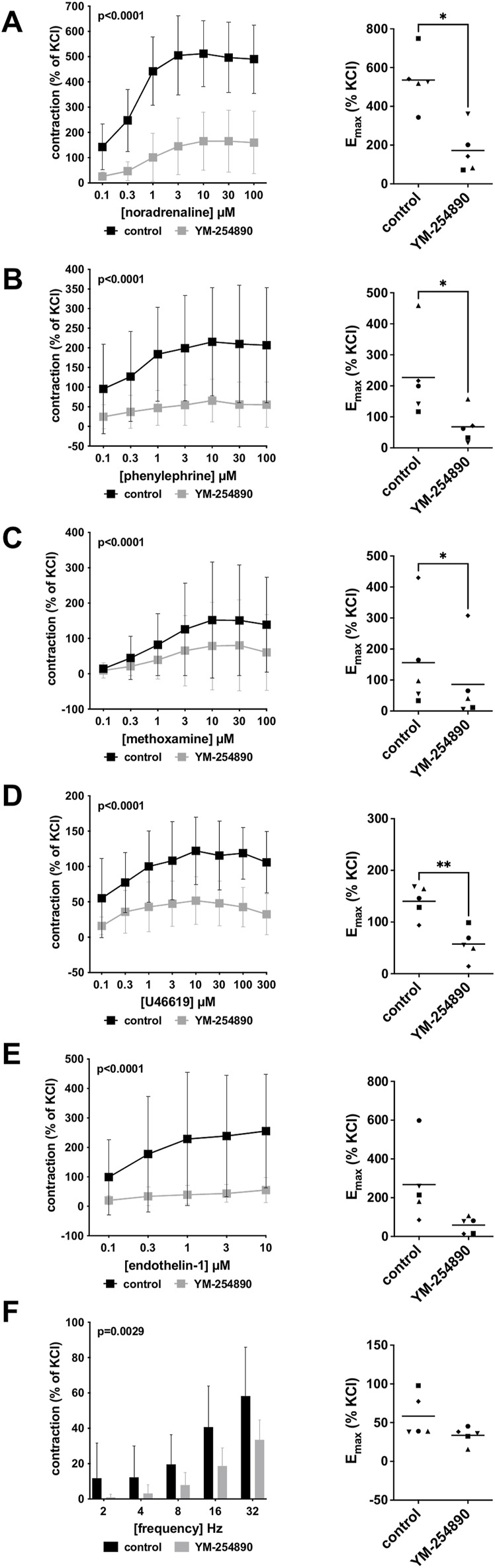
Effects YM-254890 on adrenergic and non-adrenergic, and EFS-induced neurogenic contractions of porcine renal interlobar artery tissues. Adrenergic contractions were induced by the α1-adrenoceptor agonists noradrenaline **(A)**, phenylephrine **(B)**, and methoxamine **(C)**, while non-adrenergic contractions were induced by thromboxane A2 analog (Continued) U46619 **(D)** and endothelin-1 **(E)**, and neurogenic contraction was induced by EFS **(F)**, after addition of YM-254890 (30 nM) or equal amount of solvent (DMSO) for controls (10 μl/ml). To eliminate heterogeneities due to individual variations, or varying smooth muscle content or other heterogeneities, tensions have been expressed as percentages (%) of contraction by highmolar KCl (80 mM), being assessed before application of YM-254890 or DMSO. Each experiment used strips from different animals and data are presented as means ± SD from series with tissues from *n* = 5 animals for noradrenaline **(A)**, *n* = 5 animals for phenylephrine **(B)**, *n* = 5 animals for methoxamine **(C)**, *n* = 5 animals for U46619 **(D)**, *n* = 5 animals for endothelin-1 **(E)**, and *n* = 5 animals for EFS **(F)**. Tissue from each animal was allocated to the control and drug group examined in the same experiment, resulting in paired groups and identical group sizes in each diagram. Overall *p* values reflect comparison in two-way ANOVA between treatment and control groups (*p* values for whole groups in inserts). All single Emax values from experiments are shown in scatter plots next to their corresponding agonist-response curves (*p < 0.05).

Vascular smooth muscle contraction of porcine renal arteries was induced by the adrenergic agonists noradrenaline, methoxamine and phenylephrine following incubation with YM-254890 (30 nM), or DMSO for controls. YM-254890 inhibited noradrenaline-induced contractions by 77% (68–79) at concentrations of 0.1–100 µM (overall *p* < 0.0001 for YM-254890 vs. control; Figure 5A). E_max_ was reduced by YM-254890, amounting to 536% (357–716) of KCl-induced contractions in controls and to 172% (26–318) of KCl-induced contractions after application of YM-254890 [absolute MD 364% (91–638), *p* = 0.021]. Phenylephrine-induced contractions were inhibited by 73% (71–74) at concentrations of 0.1–100 µM (overall *p* < 0.0001 for YM-254890 vs. control; Figure 5B). E_max_ was reduced by YM-254890, amounting to 227% (58–396) of KCl-induced contractions in controls and to 68% (0–136) of KCl-induced contractions after application of YM-254890 [absolute MD 159% (55–263), *p* = 0.013]. YM-254890 inhibited methoxamine-induced contractions by 48% (41–55) at concentrations of 0.1–100 µM (overall *p* < 0.0001 for YM-254890 vs. control; Figure 5C). E_max_ was reduced by YM-254890, amounting to 156% (−44 to 356) of KCl-induced contractions in controls and to 86% (57–126) of KCl-induced contractions after application of YM-254890 [absolute MD 70% (20–120), *p* = 0.018].

#### 3.4.2 Non-Adrenergic Contractions

YM-254890 inhibited contractions induced by U46619 by 61% (55–67) at concentrations of 0.1–300 µM (overall *p* < 0.0001 for YM-254890 vs. control; Figure 5D). E_max_ was reduced by YM-254890, amounting to 140% (103–178) of KCl-induced contractions in controls and to 57% (19–96) of KCl-induced contractions after application of YM-254890 [absolute MD 83% (40–126), *p* = 0.006]. Endothelin-1-induced contractions were inhibited by 81% (79–82) at concentrations of 0.1–10 µM (overall *p* = 0.0001 for YM-254890 vs. control; Figure 5E). E_max_ remained unchanged by YM-254890, amounting to 267% (24–511) of KCl-induced contractions in controls and to 59% (7–111) of KCl-induced contractions after application of YM-254890 [absolute MD 209% (−18 to 435), *p* = 0.063].

#### 3.4.3 Electric Field Stimulation-Induced Contractions

Using EFS, we investigated the effect of YM-254890 on neurogenic contractions. YM-254890 reduced EFS-induced contractions by 65% (48–82) at frequencies of 2–32 Hz (overall *p* = 0.0029 for YM-254890 vs. control; Figure 5F). E_max_ remained unchanged by YM-254890, amounting to 58% (24–93) of KCl-induced contractions in controls and to 34% (20–47) of KCl-induced contractions after application of YM-254890 [absolute MD 25% (−11 to 61), *p* = 0.129].

### 3.5 Effects of YM-254890 on Highmolar Potassium Chloride-Induced Contractions

**FIGURE 6:**
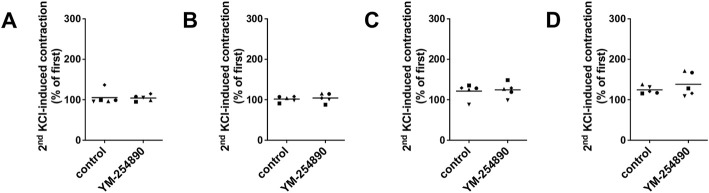
Contractions by highmolar KCl (80 mM) were induced in human prostate **(A)** and detrusor tissues **(B)**, and in porcine coronary **(C)** and interlobar arteries **(D)**. Contractions by highmolar KCl were induced by addition of 2 M KCl solution to normal Krebs-Henseleit solution in organ bath chambers (80 mM), before construction of frequency or concentration response curves (followed by washout before construction of curves, and before addition of YM-254890 or equal amount of solvent). To assess effects of YM-254890 or DMSO, contractions by KCl were induced before and after application of YM-254890 (30 nM) or equal amount of solvent (DMSO, 10 μl/ml) in the same sets of experiments, and the second KCl-induced contraction was expressed as percentage of the first KCl-induced contraction. Shown are single values from each experiment, based on double determination. In any case, the effect of KCl is graphed as means and uses individual symbols for experiments performed with the same tissue, using tissues from *n* = 5 different patients or animals for each experiment and data set.

In all tissue types, neither YM-254890 nor DMSO reduced KCl-induced contractions, compared and normalized to KCl-induced contractions assessed before application of YM-254890 or DMSO in the same tissues (Figures 6A–D). In all tissue types, KCl-induced contractions remained similar after incubation with YM-254890 or DMSO.

### 3.6 Effects of YM-254890 on Proliferation of WPMY-1 Cells

**FIGURE 7:**
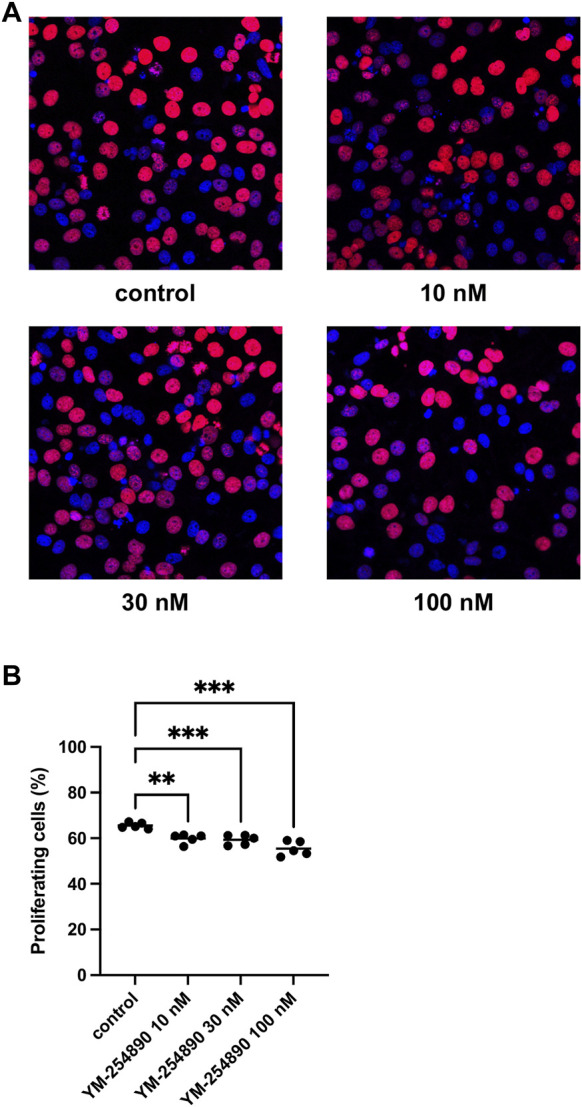
Inhibition of prostate stromal cell proliferation by YM-254890 using concentrations of 10, 30 and 100 nM. WPMY-1 cells were either allocated to a YM-254890 group, or control group containing equal amount of solvent (DMSO, 10 μl/ml), and incubated for 72 h. Shown are the percentages of proliferation (means) as scatter plots containing data of each individual experiment, which were assessed by EdU assay for each concentration, using cell cultures from *n* = 5 independent experiments for each concentration (**p < 0.01, ***p < 0.001). Proliferating cells were detected by EdU staining and counterstaining of all nuclei with DAPI, followed by fluorescence microscopy, resulting in blue-colored nuclei for non-proliferating cells and red nuclei for proliferating cells. Shown are exemplary images of cell proliferation **(A)**, and quantification of all experiments **(B)**.

YM-254890 reduced the proliferation rate in WPMY-1 cells in a concentration-dependent manner (Figures 7A,B). After exposure to YM-254890 for 72 h, the proliferation rate was reduced to 60% (57.3–62.5), 59% (56.6–62.0), and 55% (51.4–59.4) by 10, 30, and 100 nM YM-254890, respectively, while 66% (64.0–67.2) of solvent-treated cells (DMSO) showed proliferation (*p* = 0.0013, *p* = 0.0009, and *p* = 0.0007 for 10, 30, and 100 nM YM-254890 vs. control, respectively; Figure 7A).

### 3.7 Effects of YM-254890 on Colony Formation of WPMY-1 Cells

**FIGURE 8:**
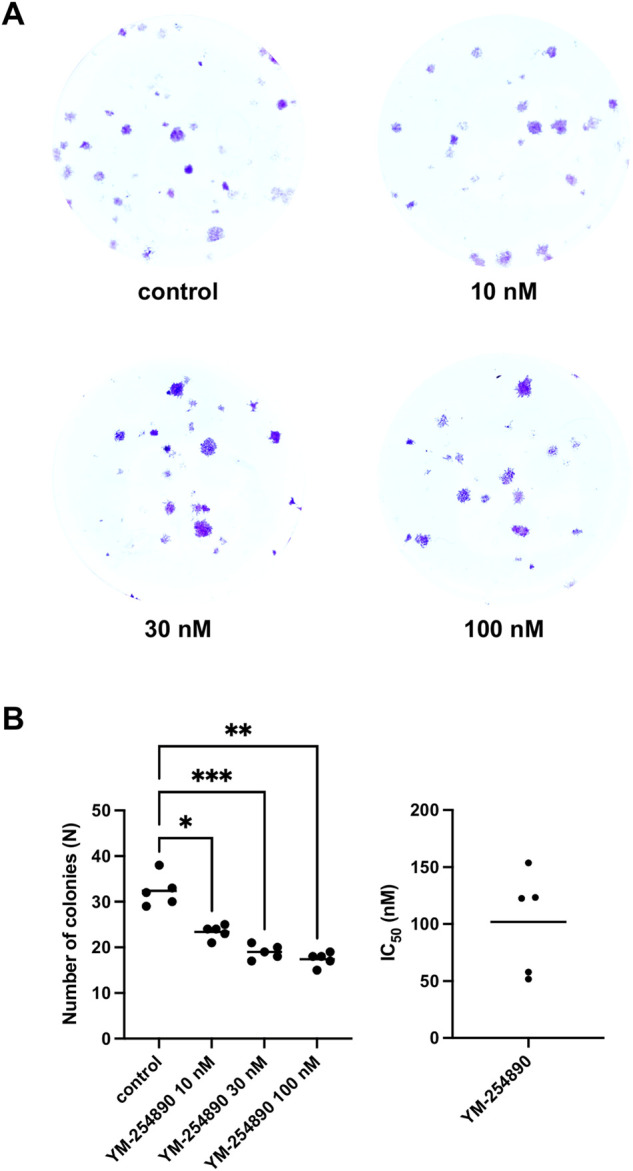
Inhibition of prostate stromal WPMY-1 cell colony formation by YM-254890. Shown is the absolute number of colonies (N) after 168 h (means) as scatter plots containing data of each individual experiment from *n* = 5 independent experiments. The cells were either allocated to a YM-254890 (10, 30, and 100 nM) or control group, with equal amount of solvent (DMSO, 10 μl/ml). Cells were subsequently incubated for an additional 168 h. Shown are exemplary images of colony formation after 168 h **(A)**, and quantification of all experiments **(B)** with corresponding IC50 values from all five experiments (right) (*p < 0.05, **p < 0.01, ***p < 0.001).

YM-254890 reduced colony formation in WPMY-1 cells in a concentration-dependent manner (Figures 8A,B). The number of colonies per well amounted to 33 colonies (28–37) for controls, and to 24 colonies (22–26), 19 colonies (17–21) and 18 colonies (16–19) with 10, 30, and 100 nM YM-254890, respectively (*p* = 0.0176, *p* = 0.0005, and *p* = 0.0015 for 10, 30, and 100 nM YM-254890 vs. control after 168 h, respectively; Figure 8B). The decline in colony formation was concentration-dependent and obviously reached a maximum at the highest applied concentration of 100 nM, what allowed curve fitting. The IC_50_ for inhibition of colony formation amounted to 102 nM (46–157).

### 3.8 Effects of YM-254890 on Viability of WPMY-1 Cells

**FIGURE 9:**
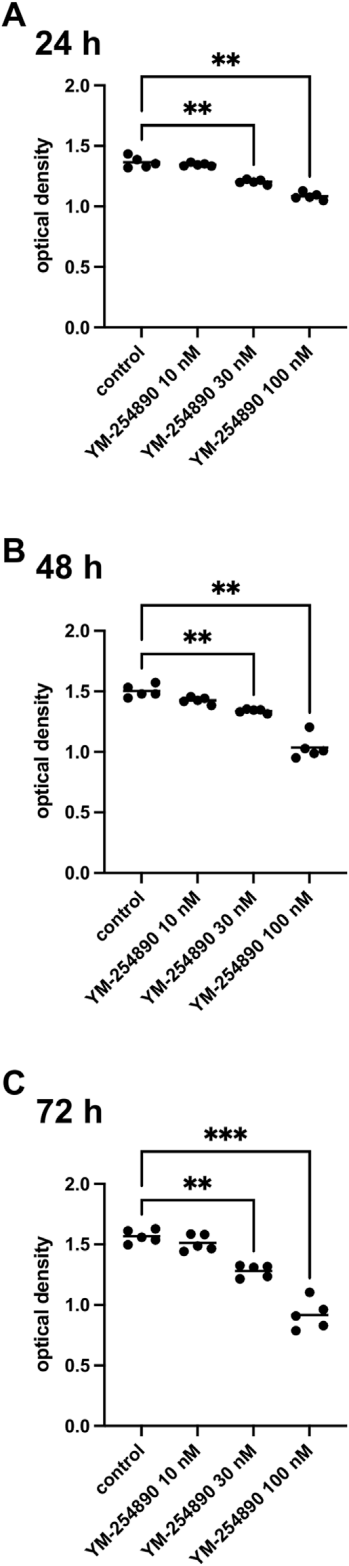
Viability of prostate stromal cells (WPMY-1) after exposure to YM-254890. Shown are values for optical density from CCK-8 assays, reflecting the amounts of viable prostate stromal cells after 24 h **(A)**, 48 h **(B)**, and 72 h **(C)** from series using cell cultures from *n* = 5 independent experiments for each indicated time. The cells were either allocated to a control (DMSO, 10 μl/ml) or YM-254890 (10, 30, and 100 nM) group and incubated for 24, 48, and 72 h (*p < 0.05, **p < 0.01, ***p < 0.001). Shown are the quantifications of all experiments as optical density (mean) as scatter plots containing data of each individual experiment **(A–C)**.

Viability of WPMY-1 was reduced following exposure to YM-254890 (10–100 nM). If referred to corresponding control, we observed decreases in viability for exposure to 100 nM YM-254890 of 21% after 24 h, [MD 0.280 in OD (0.158–0.402)], 31% after 48 h, [MD 0.466 in OD (0.225–0.707)], and 41% after 72 h, [MD 0.649 in OD (0.451–0.847)] (Figure 9). Together, the decline in viability was progressive and concentration-dependent, but remained incomplete even at the highest concentration, so that not curve fitting was performed to calculate IC_50_ values.

### 3.9 Effects of YM-254890 on Apoptosis and Cell Death of WPMY-1 Cells

**FIGURE 10:**
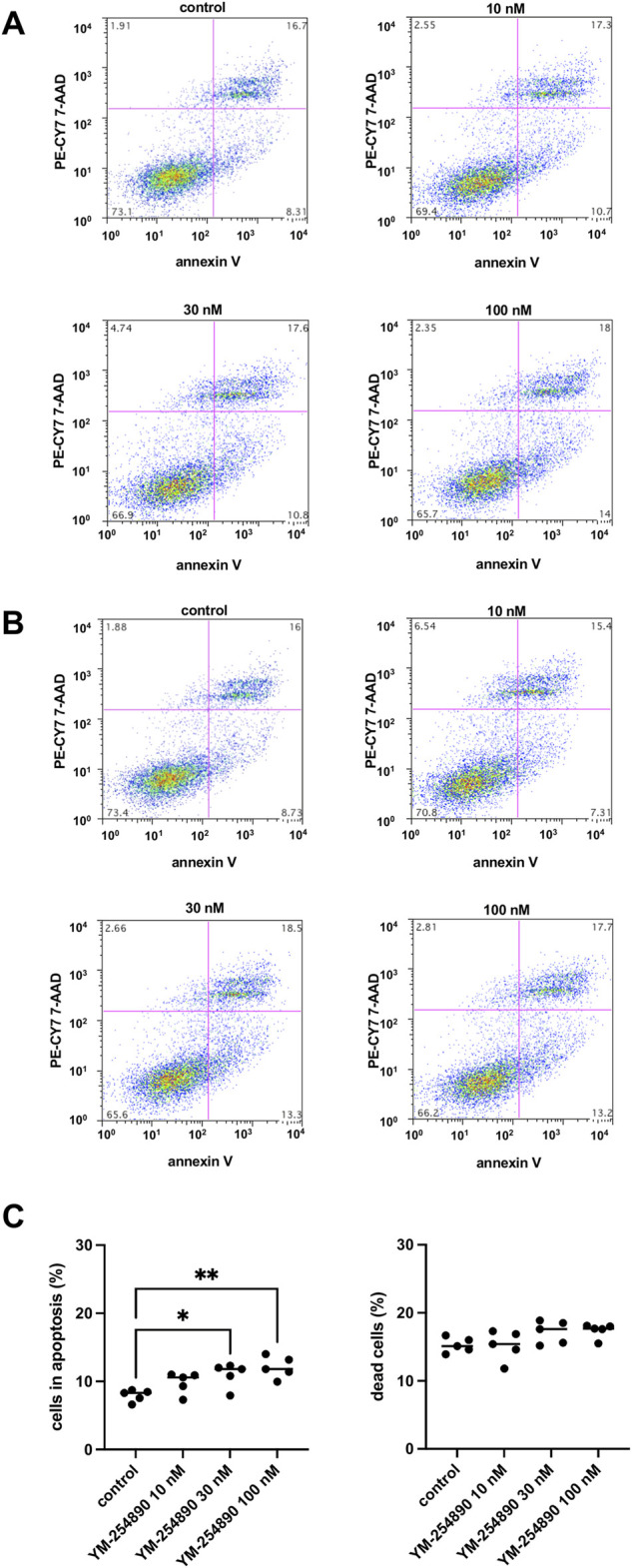
Apoptosis **(A)** and cell death **(B)** in WPMY-1 cells after stimulation with YM-254890. Flow cytometry was performed, after cells were treated for 72 h with YM-254890 (10, 30, and 100 nM) or equal amount of solvent (DMSO, 10 μl/ml) for controls. Subsequently, the numbers of cells in apoptosis (annexin V-positive, 7-AAD-negative), and of dead cells (resulting from apoptosis and/or necrosis; annexin V-positive, 7-AAD-positive) (Continued) were assessed by flow cytometry. Shown are means (percentage of cells in apoptosis, or of dead cells, referred to the number of all cells) as scatter plots containing data of each individual experiment **(C)**, and representative single experiments **(A,B)** from a series of *n* = 5 independent experiments.

At the highest concentration of 100 nM, YM-254890 increased the relative number of cells in apoptosis to 12.1% (10.2–14.0), thereby increasing apoptosis 1.5-fold compared to solvent-treated controls (*p* = 0.001 for 100 nM YM-254890 vs. control; Figure 10A). There was no significant increase in dead cells (Figure 10B,C).

### 3.10 Effects of YM-254890 on Actin Organization of WPMY-1 Cells

**FIGURE 11:**
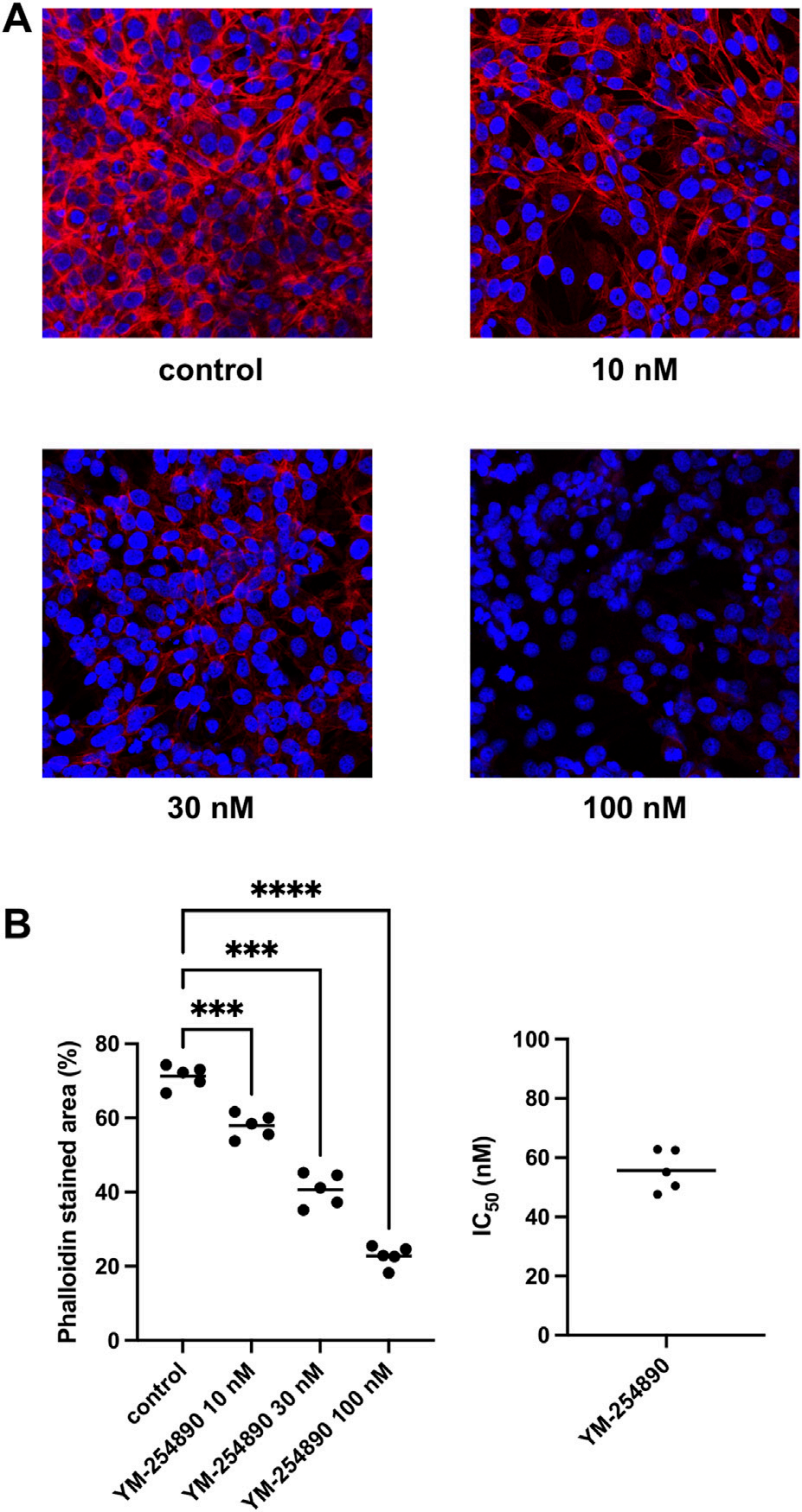
Inhibition of WPMY-1 cell actin organization by YM-254890 using concentrations of 10, 30, and 100 nM. Shown are actin filaments, visualized by phalloidin staining, after 72 h of exposure from series using cell cultures from *n* = 5 independent experiments for each panel. The cells were either allocated to a control (DMSO, 10 μl/ml) or YM-254890 group and incubated for 72 h. Actin filaments were visualized by phalloidin staining and fluorescence microscopy, while the nuclei were visualized using DAPI staining. IC50 values were calculated by curve fitting of each single experiment. Shown are representative single experiments from series of *n* = 5 independent experiments for each panel **(A)**, and quantification of all experiments as means in scatter plots containing data of each individual experiment quantified as percentage (%) of phalloidin-stained area (***p < 0.001, ****p < 0.0001) after 72 h **(B)** with corresponding IC50 values from all five experiments (right).

Actin filaments in solvent-treated control WPMY-1 cells were arranged to bundles of long and thin protrusions, and elongations from adjacent cells were overlapping each other (Figure 11). YM-254890 (10–100 nM) caused concentration-dependent degradation of actin filaments after incubation for 72 h, resulting in a rounded cell shape without any protrusions. YM-254890 caused regression of phalloidin-stained areas from 71% (67–75) in controls to 23% (19–26) 100 nM, thereby reducing actin formation by a MD of 48% (42–55) (*p* < 0.0001 for 100 nM YM-254890 vs. control; Figure 11). The IC_50_ for breakdown of polymerized actin by YM-254890 amounted to 56 nM (47–64).

## 4 Discussion

Contractile smooth muscle receptors may be coupled to Gα_q_ or Gα_12/13_ subunits, or both, but only little is known about the specificities, as to which receptor prefers which Gα, or whether Gα_q_ is coupled to all of them, and about differences in G protein coupling of the same receptor in different types of smooth muscle (Somlyo and Somlyo, 2000; Hennenberg et al., 2014). With YM-254890, a small molecule inhibitor with presumed specificity for Gα_q_ has become available (Peng et al., 2021). Our present findings suggest ubiquitous susceptibility to YM-254890 of agonist-induced smooth muscle contractions in four different tissues, including α_1_-adrenergic contractions in prostate tissues and renal arteries, cholinergic contractions in detrusor tissues and coronary arteries, and thromboxane- and endothelin-1-induced contractions in all four tissues. Even though comparisons across different series of experiments underlie limitations, some differences between different tissues are conspicuous. Differences became essentially obvious between prostate smooth muscle tissues, where contractions by all agonists were nearly completely inhibited by YM-254890, and detrusor smooth muscle tissues, where contractions were apparently only partly inhibited. In WPMY-1 cells, YM-254890 showed effects on growth-related functions, which were clearly detectable, but clearly limited as well, and probably too weak to translate to effects on prostate growth *in vivo*. Together, our findings may be discussed in the context of basic smooth muscle physiology but may also add to understanding of LUTS in urology, where experimental findings previously suggested promotion of growth by α_1_-adrenoceptors in prostate cells, while α_1_-blockers did not reduce prostate volume in patients with BPH (Kyprianou et al., 1998; Kyprianou et al., 2000; Glassman et al., 2001; Chagas-Silva et al., 2014; Nascimento-Viana et al., 2019; Gravas et al., 2021).

YM-254890 is a cyclic depsipeptide compound, which was originally isolated from *Chromobacterium* sp. and claimed as a specific Gα_q_ inhibitor (Taniguchi et al., 2003; Takasaki et al., 2004). YM-254890 inhibits signaling mediated by Gα_q_-coupled receptors, with a high potency and an IC_50_ around 30 nM, by blocking guanosine triphosphate (GTP) exchange from guanosine diphosphate (GDP), thereby preventing Gα_q_ activation (Nishimura et al., 2010). Based on previous findings confirming that 30 nM YM-254890 abolished UTP-activated P2Y_2_ receptor-mediated Ca^2+^ signaling and ERK1/2 phosphorylation in human coronary endothelial cells, we applied YM-254890 in a concentration of 30 nM in organ bath experiments, and concentrations ranging from 10 to 100 nM in cell culture experiments (Peng et al., 2021). However, YM-254890 was also shown to inhibit G_s_ protein-mediated cyclic adenosine monophosphate (cAMP) production in the same study with a calculated IC_50_ of 30 nM (Peng et al., 2021).

Agonist-induced smooth muscle contractions are caused by activation of 7-transmembrane receptors, coupled to heterotrimeric G proteins each including a Gα_q_ and/or Gα_12/13_ subunit (Somlyo and Somlyo, 2000; Hennenberg et al., 2014). Receptor activation results in subsequent activation of intracellular post-receptor signaling by Gα subunits, ultimately leading to contraction by accumulation of phosphorylated myosin light chains (MLC) through activation of MLC kinase or inactivation of MLC phosphatase (Somlyo and Somlyo, 2000; Hennenberg et al., 2014). In fact, contractile receptors in all smooth muscle types share three prototypic intracellular signaling pathways, which are believed to be activated in parallel, including inositol 1,4,5-trisphosphate (IP_3_)/calcium-dependent mechanisms of MLC kinase activation, and MLC phosphatase inactivation by diacylglycerol- (DAG)-mediated protein kinase C activation and by RhoA/Rho kinase (Somlyo and Somlyo, 2000; Hennenberg et al., 2014). IP_3_ and DAG are generated by phosphatidylinositol 4,5-bisphosphate hydrolysis by phospholipase C (PLC), which is typically activated by Gα_q_ in smooth muscle cells (Frazier et al., 2008; Black et al., 2016). Based on exemplary findings, activation of RhoA has been attributed/related to Gα_12/13_ (Gohla et al., 2000; Black et al., 2016). However, whether proposed concepts of an Gα_q_/PLC axis and Gα_12/13_ axis can be in fact generalized to all contractile receptors and to all types of smooth muscle cells, remains uncertain and to be proven (Somlyo and Somlyo, 2000; Black et al., 2016). In fact, the extent of RhoA activation was found to differ with contractile agonists (Sakurada et al., 2001). While thromboxane receptors were supposed to couple preferentially to Gα_12/13_, endothelin-1 was presumed to use Gα_q_ and Gα_12/13_ for signal transduction (Offermanns et al., 1994). On the other hand, Gα_q_ may activate RhoA as well, together reflecting the highly anecdotic and exemplary character of available findings (Momotani et al., 2011). Thus, the preference of different contractile receptors, or of different smooth muscle types for either Gα_q_ or Gα_12/13_ still represents a central question in smooth muscle physiology. Here, we examined effects of YM-254890 on agonist-induced and neurogenic contractions in four smooth muscle-rich tissues, including human prostate and detrusor tissues, and porcine renal and coronary arteries.

Obviously, contractions of prostate and detrusor tissues showed divergent susceptibilities to YM-254890, as contractions by all examined agonists were only partly inhibited in detrusor tissues, but virtually completely in prostate tissues. In the light of this observation, it may be speculated that contractile receptors are coupled to Gα_q/11_ in human prostate smooth muscle cells, but to lesser extent (e.g., only to parts of receptor populations) in human bladder smooth muscle. In fact, this is in line with findings suggesting that human bladder smooth muscle contraction by muscarinic receptors does not (or not necessarily) involve activation of PLC (Frazier et al., 2008). Activation of PLC by contractile receptors is imparted by Gα_q/11_, which is considered a prototypical pathway of agonist-induced smooth muscle contraction, with the exception of M3 receptors in bladder smooth muscle (Frazier et al., 2008). As inhibitions of non-cholinergic contractions in our detrusor tissues remained incomplete, it may be concluded that this phenomenon is not limited to M3 receptors but includes other contractile receptors in bladder smooth muscle as well. However, the previously noted special role of bladder M3 receptors is apparently not bladder-specific, as we observed full inhibition of cholinergic contractions in coronary arteries. Thus, contractile receptors seem to be universally coupled to Gα_q/11_ in the human prostate, while the contribution of Gα_q_ to agonist-induced contractions in the human detrusor is lower.

In fact, it has been suggested that contractions by cholinergic agonists may differ from those by other agonists. In bladder and airway smooth muscle tissues, β-adrenoceptor agonists exhibited lower efficacy as relaxing compounds if tissues were precontracted by muscarinic agonists, compared to precontraction with other agents, including high-molar KCl (Dale et al., 2014). At a clinical level, this stands in unison with the Symphony trial (NCT01340027), which showed a positive impact on patients treated for OAB with combinations of antimuscarinic agents and the β_3_-agonist mirabegron, decidedly advocating the combination use of different drug classes (Abrams et al., 2015). While precise mechanisms underlying the unique characteristics of muscarinic contractions remain unclear, PLC- and thus, Gα_q_-independent components of bladder smooth muscle contraction are well known and may include voltage-operated calcium channels and Rho kinase (Frazier et al., 2008). This is consistent with the findings by Phelps and colleagues, who used porcine urothelium and lamina propria, and observed partial reduction of GPCR-mediated contraction by nifedipine, which was maintained in a Ca^2+^-free environment, suggesting internal stores of Ca^2+^, activated by G_q/11_ receptor proteins in those tissues (Phelps et al., 2022). Notably for YM-254890, inhibitory effects on such a wide range of contractile agonists through the specific Gα_q/11_ inhibitor are novel and may indeed be due to shared post-receptor signaling in smooth muscle contractile receptors.

Regarding renal and coronary arteries, certain differences exist as well: while α_1_-adrenergic contractions are known from most vessel types but do not occur in coronary arteries, cholinergic contractions typically do not occur in renal interlobar arteries (Moreland and Bohr, 1984; Li et al., 2021). Accordingly, we examined cholinergic contractions only in coronary arteries. α_1_-Adrenergic contractions are shared by vascular and prostate smooth muscle, but showed slightly divergent reactions to YM-254890. Contractions by all three α_1_-agonists appeared to be inhibited stronger in prostate tissues than in interlobar arteries, where they were partly or incompletely inhibited. The same applied to U46619-induced contractions. Whether this reflects differences in smooth muscle tissue types, or involvement of G_s_ protein-coupled post receptor signaling pathways (prostate vs. vascular, e.g., α_1A_ vs. α_1D_ subtypes of α_1_-adrenoceptors), or species-dependent differences between smooth muscle tissues from humans and pigs, cannot be clarified on the basis of our data.

However, the inhibitory effect of YM-254890 in our study eems fairly Gα_q_-specific, while an involvement of Gα_s_ inhibition appears highly unlikely. While YM-254890 was recently also reported as a Gα_s_ inhibitor, the effects we observed may not be due to inhibition of Gα_s_-mediated cAMP production (Peng et al., 2021). Thus, if YM-254890 inhibited Gα_s_ in our tissues, we would not expect inhibition of contraction, but increased contraction, considering that Gα_s_ causes cAMP production, finally resulting in smooth muscle relaxation. In line, we observed that YM-254890 was not able to inhibit KCl-induced contractions in any of the examined tissues, which should occur, if the compound inhibited Gα_s_ and subsequent cAMP production Thus, it is fairly possible to support specificity of YM-254890 to Gα_q_.

Limitations of our study certainly include variability of data in organ bath experiments, which was most pronounced in our experiments with neurogenic detrusor contractions. Still, several factors may generally account for high variability between patients. Tissue heterogeneity is particularly high in human tissues, due to individual variations (i.e., larger than in standardized laboratory animals), age (i.e., greater variability and span than in standardized laboratory animals), or even different pathological backgrounds, e.g., BPH, OAB, or mixed LUTS. To reduce limitations, we verified the neurogenic origin of EFS-induced detrusor contractions, by showing they were fully sensitive to TTX, and referred all contractions to KCl.

In parallel to prostate smooth muscle tone, static obstruction as a direct consequence of prostatic stromal cell growth may contribute to peri-urethral compression in BPH as well and presents an important target for drug treatment (Oelke et al., 2013; Hennenberg et al., 2014). 5-ARI decrease prostate size, although by no more than 25% and are applied to prevent disease progression and complications (Fusco et al., 2018; Gravas et al., 2021). Cytokines, growth factors and in particular hormones (e.g., androgens, estrogens) have a decisive influence on prostate growth, and molecular mechanisms at the level of intracellular signaling are an important and clinically relevant focus of interest (Sampson et al., 2007; Timms and Hofkamp, 2011). Based on experimental studies, it has been repeatedly suggested, that α_1_-adrenoceptors mediate prostatic growth. After chronic administration of phenylephrine *in vivo*, rats and mice develop hypertrophic and dysplastic changes in the prostate (Golomb et al., 1998; Marinese et al., 2003). Similarly, findings from sympathectomized rats suggested connections between prostatic growth and sympathetic innervation via α_1_-adrenoceptors (McVary et al., 1994). In fact, prostatic α_1_-adrenoceptors induce proliferation, suppress apoptosis, and activate growth-promoting kinases in prostate cells, including ERK1/2 and others (Kyprianou et al., 1998; Kyprianou et al., 2000; Glassman et al., 2001; Kanagawa et al., 2003; Maroni et al., 2004; Hennenberg et al., 2014). Together, these findings raised the assumption that treatment with α_1_-adrenoceptor antagonists in BPH may prevent prostatic growth. In contrast, there is large consensus from numerous clinical studies and decades of clinical experience, that α_1_-blockers do not reduce prostate volume in patients with LUTS/BPH (Roehrborn, 2006; Roehrborn et al., 2008; Roehrborn et al., 2010; Gravas et al., 2021).

This persistent controversy prompted us to examine effects of YM-254890 on growth-related functions of WPMY-1 cells. In previous studies, YM-254890 abolished ERK1/2 activation in exposed tissues, probably by inhibition of Gα_q/11_ (Peng et al., 2021). We observed that YM-254890 reduced proliferation rate, growth in colony formation assays and viability in WPMY-1 cells, while the number of apoptotic cells was increased by YM-254890. These effects occurred within a concentration range of 10–100 nM. Data of some series allowed curve fitting, which pointed to IC_50_ values for YM-254890 in nanomolar ranges, which are in line with previous values for inhibition of Gα_q/11_ by YM-254890 (Peng et al., 2021). While the observed effects may be due to anti-proliferative effects facilitated through an ERK1/2 inhibition by YM-254890, we recognize that cellular toxicity of YM-254890 may have contributed as well. However obvious, effects on growth-related functions of stromal cells were clearly limited and small as well, and *in vivo* translation may not be possible based on these experiments. However, this may explain why previous studies in experimental models suggested α_1_-adrenergic growth of prostate cells, but α_1_-blockers did not affect prostate size *in vivo* (Oelke et al., 2013; Hennenberg et al., 2014).

Actin polymerization and correct filament organization are required for any type of smooth muscle contraction. YM-254890 quantitatively and qualitatively altered actin organization in a concentration-dependent manner. Together with the completely abolished adrenergic contractions in human prostate smooth muscle tissues, we assume that Gα_q/11_ and its upstream receptors promote contraction not only by MLC phosphorylation but also by actin-dependent mechanisms, at least in prostate smooth muscle. Considering that anticontractile effects were limited in detrusor tissues, it appears possible that the role and contribution of Gα_q/11_-dependent actin organization differs between smooth muscle-rich organs. While YM-254890 seems to marginally increase apoptosis, there was otherwise no increase in undirected cell death. Consequently, decrease in viability observed in CCK-8 assays may not be attributed to cell death due to cytotoxicity, which is why we performed flow cytometry. Thus, disruption of actin organization may be due to Gα_q/11_-specific action of YM-254890, as proliferation was disproportionately reduced in a very limited manner only. Divergent incubation periods in the organ bath (30 min) and in cell culture (72 h) certainly pose another limitation of our study, as it remains unclear whether disruption of actin filament organization was due to specific inhibition of G_q_ protein signaling, or if toxicity may have contributed. Furthermore, we do not know if contractile agonists would have had an effect on WPMY-1 cells in cell culture experiments.

Medical therapy for LUTS suggestive of BPH includes α_1_-adrenoceptor antagonists for rapid relieve of voiding symptoms by inhibiting prostate smooth muscle contraction, and 5-ARI for long-term reduction of prostate size, and to prevent disease progression (Fusco et al., 2018; Gravas et al., 2021). Medical therapy for OAB is primarily based on anticholinergic medications for storage symptoms. However, discontinuation rates due to limited efficacy and disproportional side effects range around 80% within 12 months (Sexton et al., 2011). Meanwhile, the limited efficacies have been explained by non-adrenergic prostate smooth muscle contractions and by non-cholinergic detrusor contractions (thromboxane, endothelin), and OAB-induced detrusor contractions are supposed to be non-cholinergic in origin. Nevertheless, non-adrenergic and non-cholinergic contractions in the lower urinary tract have hardly been taken into account in basic research so far. With the β_3_-adrenergic agonist mirabegron, the first alternative to anticholinergics was introduced. However, its efficacy does not exceed that of established antimuscarinics, so that medical treatment of storage disorders still represents a significant problem (Dahm et al., 2017). As regulation of prostatic and bladder smooth muscle tone remains a crucial target in LUTS, BPH and OAB, increased understanding of molecular mechanisms may be beneficial for future medical treatment options (McNeal, 1990). Thus, fundamental understanding of underlying mechanisms in smooth muscle contraction, including organ-specific characterization of contractile receptors, may not only be relevant for the lower urinary tract, but for the cardiovascular system and other smooth muscle-rich organs as well.

## 5 Conclusion

Our findings suggest shared intracellular signaling by Gα_q_-coupled contractile receptors in multiple smooth muscle-rich organs, but also point to possible differences. α_1_-Adrenergic contractions in prostate smooth muscle and renal arteries, cholinergic contractions in detrusor tissues and coronary arteries, and thromboxane- and endothelin-induced contractions in each of these tissues showed ubiquitous susceptibility for YM-254890. Differences were most obvious between prostate smooth muscle, where contractions by all agonists were nearly completely inhibited, and detrusor smooth muscle, where contractions by all agonists were inhibited only partly. YM-254890 affected growth-related functions in prostate stromal cells, which were clearly detectable, but clearly limited as well. Our findings significantly add to improved understanding of basic smooth muscle physiology but may also be applicable in the context of LUTS treatment in urology, where previous findings suggested promotion of growth by α_1_-adrenoceptors in prostate cells, while α_1_-blockers do not reduce prostate volume in patients with BPH. Together this may aid in developing new pharmaceutical targets for LUTS and antihypertensive medication in the future.

## Data Availability

The raw data supporting the conclusions of this article will be made available by the authors, without undue reservation.
