# Supplementary figures and images for: Inhibition of Human Prostate and Bladder Smooth Muscle Contraction, Vasoconstriction of Porcine Renal and Coronary Arteries, and Growth-Related Functions of Prostate Stromal Cells by Presumed Small Molecule Gαq/11 Inhibitor, YM-254890
Source: Front Physiol. 2022 May 23;13:884057. doi: 10.3389/fphys.2022.884057 (PMC9168773; doi:10.3389/fphys.2022.884057)

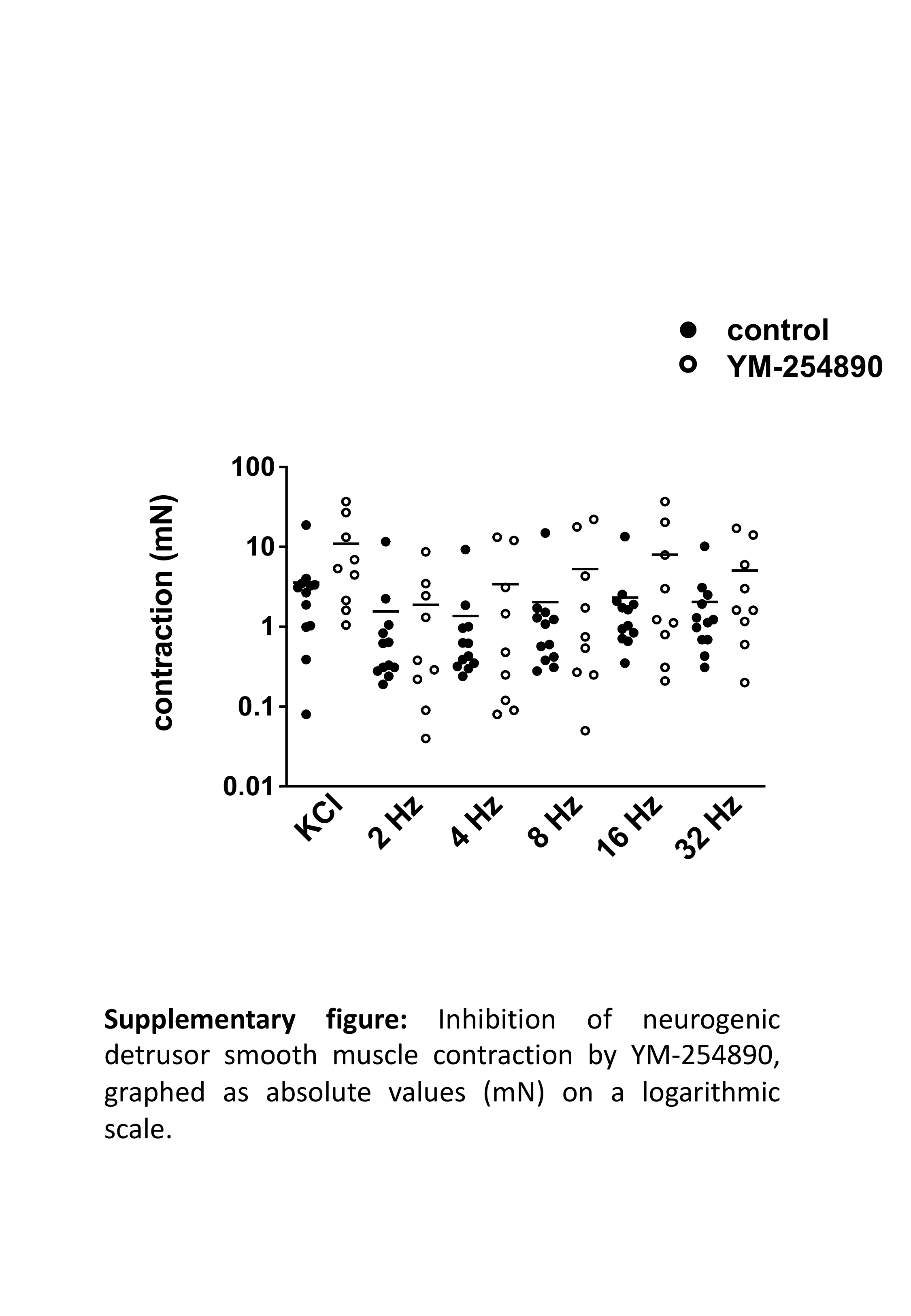

Supplement: Supplementary file 1 [file Image1.TIFF]
